# Wideband RCS Reduction Using Coding Diffusion Metasurface

**DOI:** 10.3390/ma12172708

**Published:** 2019-08-23

**Authors:** Luqman Ali, Qinlong Li, Tayyab Ali Khan, Jianjia Yi, Xiaoming Chen

**Affiliations:** 1 School of Electronics and Information Engineering, Xi’an Jiaotong University, Xi’an 710049, China; 2 Department of Electronic Engineering, City University of Hong Kong, Hong Kong SAR 999077, China; 3 Key Laboratory of Integrated Services Networks, Xidian University, No.2 South Taibai Road, Xi’an, Shannxi 710071, China

**Keywords:** radar cross-section (RCS), optimization algorithm, scattering field, metasurface

## Abstract

This paper presents a radar cross-section (RCS) reduction technique by using the coding diffusion metasurface, which is optimised through a random optimization algorithm. The design consists of two unit cells, which are elements ‘1’ and ‘0’. The reflection phase between the two-unit cells has a 180° ± 37° phase difference. It has a working frequency band from 8.6 GHz to 22.5 GHz, with more than 9 dB RCS reduction. The monostatic RCS reduction has a wider bandwidth of coding diffusion metasurface as compared to the traditional chessboard metasurface. In addition, the bistatic performance of the designed metasurfaces is observed at 15.4 GHz, which shows obvious RCS reduction when compared to a metallic plate of the same size. The simulated and measured result shows the proficiency of the designed metasurface.

## 1. Introduction

Over the past decade, remarkable research attention is paid to achieve target hiddenness or transparency of electromagnetic waves (EM). It has a low profile, less weight, and it can be used to control the scattering of EM waves. The metasurface has been used to obtain the better radar cross-section (RCS) reduction by manipulating the EM waves. Different techniques have been proposed for RCS reductions in the literature, such as the active or passive cancellation and radar absorbing materials. Various solutions have been analyzed in these RCS reduction methods [1,2,3,4,5]. 

The RCS reduction can be achieved while using metasurfaces consisting of equivalent perfect electric conductors (PEC) and artificial magnetic conductors (AMC) unit cells. However, it is very difficult to design a wideband AMC unit cell. In [6], a Jerusalem AMC unit cell is used to obtain 10 dB RCS reduction over a relative bandwidth of 41%. In [7,8], the twin-AMC cells are used to get 10 dB RCS reduction, where the reflection phase of the two-unit cell is 180° ± 37°. The AMC unit cell is used to define the metasurface for wider bandwidth. In [9], the chessboard metasurface that is composed of four E-shape and arrow type AMC cell achieves 10 dB RCS reduction for a bandwidth of 85%. The chessboard metasurface backscatters the reflecting energy into four different directions, which makes it less operative for large incident angles. It is highly desirable to design a metasurface with better RCS reduction and less complexity. 

In 2007, Paguay et al. used a combination of an AMC and PEC arrangement in a metasurface that supplies the pattern to a scattering metasurface with non-specular behaviour [10]. The reflected energy scattered into four lobes. However, the RCS reduction is maximum in the normal direction. Later, in 2013, Zhao used two different AMC structures to minimize the RCS in a wideband [11]. A better idea of the coding metasurface was proposed by Cui in 2015, which provides a path to manipulate EM waves in a more sophisticated way. It can be used in various codes, programmable field gate array (FPGA), and in switching metasurface [12]. Two types of AMC cells in the Tri shaped format were analyzed for RCS reduction in a specific direction is used in [13] with eight lobes. Chan et al. used the same unit cell, but the size of the cell changes randomly [14]. Wang proposed a low-scattering metasurface that is based on the far-field scattering while using optimization algorithm [15]. A terahertz metasurface of the single medium is planned to show very low reflection across a wider spectrum and incident angles [16]. A broadband metasurface with multi-bit coding was employed in the terahertz metasurface [17].

The coding diffusion metasurface has a low RCS based on the optimization algorithm [15]. It is more accurate on the basis of manipulating electromagnetic waves and designing various coding sequences when compared to the traditional chessboard metasurface [18,19,20]. All of the techniques referred to demonstrate the validity of assembling EM material, because it can improve the metasurface scattering performance. The mitigation of in-band RCS is its main concern, while out-of-band RCS is also a key frequency locality for radar recognition [21,22]. The metasurface has the capability to manipulate polarization [21,22,23,24] with better performance to achieve RCS [25,26,27,28]. In [29], two square rings were used with different outer edges to obtain wideband RCS. In [30], an efficient strategy is developed to design metasurface by using a combination of diffuse reflection and dispersion theory pattern remodeling method, together with a genetic algorithm (to optimize unit sequences of non-periodic random metasurface structure cells). 

**Figure 1:**
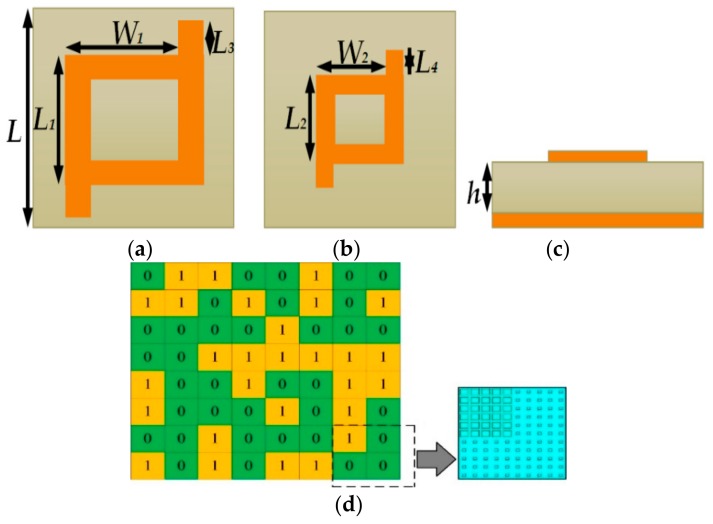
(**a**) Unit cell “1”; (**b**) Unit cell “0”; (**c**) Unit cell side view; and, (**d**) Description of the detailed configuration of two elements “0” and “1” through an optimization algorithm.

The two-unit cells 1 and 0 are designed for the coding diffusion metasurface. To get 10 dB RCS reduction, the reflection phase of the two-unit cell must be 180° ± 37°, which clearly characterises the designed unit cell, as shown in Figure 1, which operates from 8.6 to 22.5 GHz. By choosing the sequence of the unit cell uniquely while using an optimization algorithm, ultra-wideband RCS reduction is obtained. The pattern of elements and array factors are accessing, while a randomly round number is used in the optimization algorithm to obtain a two-dimensional (2-D) code. The unit cells are arranged according to the 2-D code. While using the coding diffusion metasurface, the total bandwidth is achieved from 8.6 GHz to 22.5 GHz with >9 dB RCS reduction as compared to the metal of the same size.

This document has been organized as given. In Section 2, the unit cell is designed and the phase difference is calculated from the reflection phase of the unit cell “1” and “0”. Section 3 includes analysis and simulation of the coding diffusion metasurface, which is optimized through a random optimization algorithm. Section 4 defines the fabrication and measurement of coding diffusion metasurface and the conclusion is presented in Section 5.

## 2. Unit Cell Design

The composed metasurface unit cell consists of two layers. In between these two copper layers, a substrate is inserted with a dielectric constant of 2.65 and a tangent loss of 0.001. The thickness of a substrate is *h* = 3 mm. The upper layer is a rectangular patch ring shaped structure, as shown in Figure 1, respectively. Figure 1d shows the positions of the super cell units 1 and 0. The super cells 1 and 0 both consist of 5 × 5 unit cells. The position of the super cell is determined by the optimization algorithm, which randomly generates the two-dimensional code. This algorithm is used to reflect the incident wave. It is optimized using a random optimization algorithm which manages the variation of theta and phi in such a random way that it disperses the incident wave in different directions to achieve wideband RCS reduction. Obtaining a reflection phase difference between the two units cell around 180° ± 37° is important to obtain large bandwidth. The parameters of the unit cells 1 and 0 are shown in Figure 1, where *L* = 6.6 mm, *L*_3_ = 0.3 mm and *L_4_* = 0.15 mm, respectively. 

**Figure 2:**
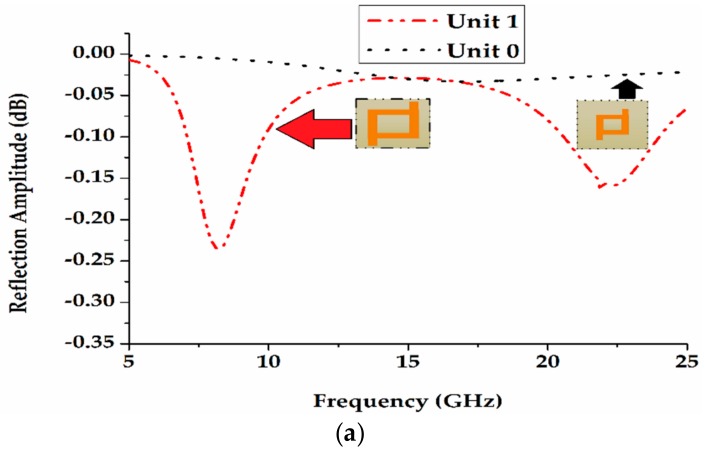

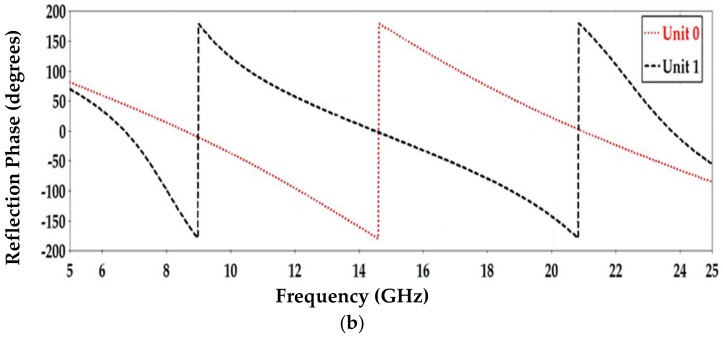
(**a**) Reflection magnitudes of unit cells ‘1’ and ‘0’; (**b**) Reflection phase of the unit cells ‘1’ and ‘0’.

**Figure 3:**
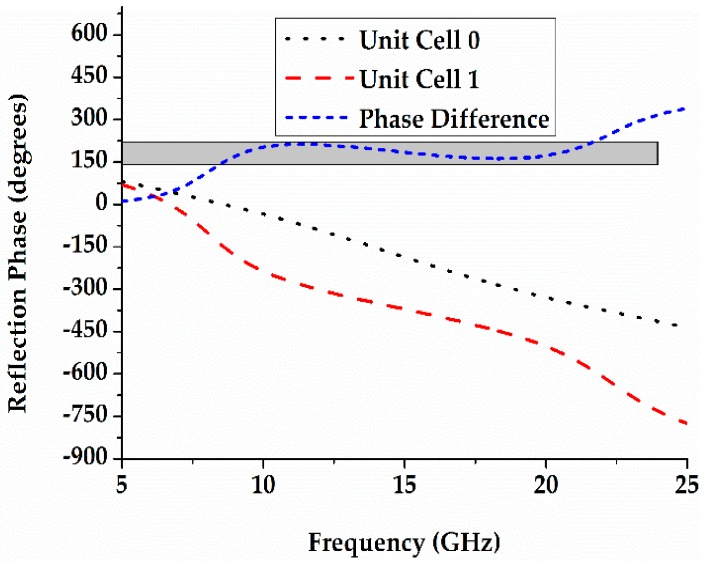
The reflection phase difference of unit cell “1” and “’0”.

The unit cell has width *W*_1_ = 4.9 mm and length *L*_1_ = 5.1 mm for the unit cell ‘1’, and width *W*_2_ = 2.05 mm and length *L*_2_ = 2.13 mm for the unit cell ‘0’, as shown in Figure 1, respectively. The two-unit cells reflection phase and reflection amplitude as a function of frequency are shown in Figure 2. It is clearly noticed that the difference between the reflection phases of two unit cells is 180° ± 37° from 8.6 GHz to 22.5 GHz, as shown in Figure 3. In this way, the unit cells are chosen to obtain a low RCS with wider bandwidth.

The reflection coefficients of unit cells 1 and 0 can be shown as a function of frequency in Figure 2. The unit cell 0 reveals 0° reflection phase from 8.6 GHz to 21.3 GHz, while the unit cell 1 reflection phase 0° exhibit from 6.7 GHz to 14.4 GHz. Figure 2 shows that the reflection phase difference alters around 180° in a wide bandwidth. The bandwidth is defined over the frequency range, where the variation of the reflection angle is within ±37°. It ranges from 8.6 GHz to 22.5 GHz (corresponding to a fractional bandwidth of 92%), as shown in Figure 3.

The Unit cell 1 is defined as an element with a π phase response and unit cell 0 is defined as an element with a zero phase response. In such a way, the “1” and “0” phase response can be elaborated as *Δϕ = nπ*. The element pattern is described by the following equation.

(1) $$E_{P}=\cos\theta$$

The eight elements of linear scattering field array can be expressed as:

(2) $$F\left(\theta \right)=E_{P}\cdot A_{F}=\cos\theta \cdot \overset{8}{\underset{c=1}{\sum }}A_{c}\cdot e^{j\frac{2\pi }{\lambda }\;x_{c}\sin\theta \cdot e^{j\varphi c^{\pi }}}\;\mathrm{and}\;x_{c}=\left(c-0.5\left(C+1\right)\right)d,\;A_{c}=1$$

The maximum value of the scattering field is employed as a fitness function to operate the scattering waves, which are given as

(3) $$FITNESS=\mathrm{MAX}F\left(\theta \right)$$

The fitness function must be minimized by using an algorithm to generate the random round set of a binary sequence.

(4) $$F\left(\theta ,\varphi \right)=E_{P}\cdot A_{F}=\cos\theta \cdot \overset{8,8}{\underset{c=1,d=1}{\sum }}A_{cd}\cdot e^{j\left(\frac{2\pi }{\lambda }\;x_{c}\sin\theta \cos\varphi +\frac{2\pi }{\lambda }y_{d}\sin\theta \sin\varphi \right)\cdot e^{j\varphi_{c,d}\pi }}$$

where $y_{d}=\left(d-0.5\left(C+1\right)\right)d,\;A_{c}=1$.

**Figure 4:**
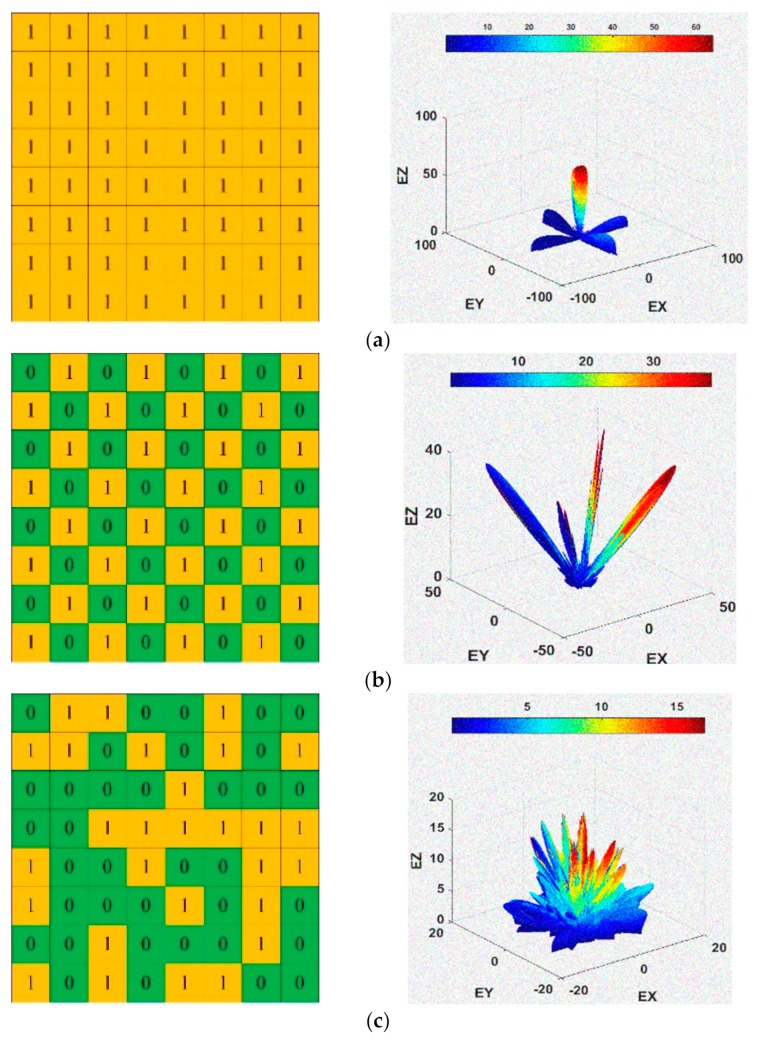
(**a**) Scattering field and two-dimensional code of metal; (**b**) Scattering field and two-dimensional code of chessboard metasurface; and, (**c**) Scattering field and two-dimensional code of coding diffusion metasurface.

Figure 4 presents the scattering field level of metal, chessboard, and coding diffusion metasurface. When the optimisation algorithm is applied, the incident beam is reflected into different directions and a wider bandwidth is obtained as compared to other surfaces. The optimisation algorithm manages the variation of theta and phi in such a random way that it disperses the incident wave in different directions, which clearly characterises the property of diffusion. A metal surface consists of the only single lobe with a scattering level of 64 dB. The chessboard metasurface has four lobes and the coding diffusion metasurface has eight lobes. The scattering field level is reduced for the coding diffusion metasurface and the incident wave disperses more as compared to other surfaces. As a result, a wider bandwidth is achieved.

**Figure 5:**
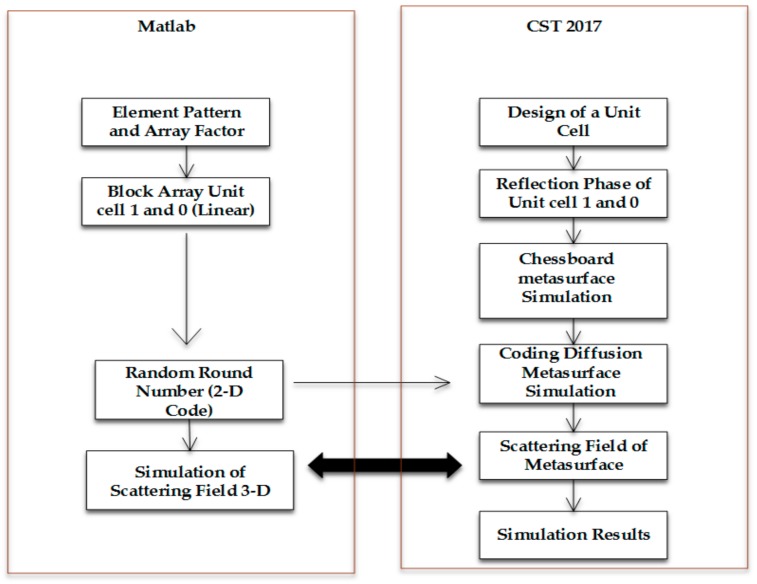
Flow chart of coding diffusion metasurface.

Figure 5 shows the flow chart of the design of the coding diffusion metasurface. CST microwave studio 2017 (CST, Darmstadt, Germany) and Matlab 2017a (Mathwork, New York, NY, USA) are both used in the flow chart. CST is used to design the rectangular patch ring and the designed metasurface. Matlab is used for optimising the element pattern.

## 3. Simulation and Analysis of Coding Metasurface

**Figure 6:**
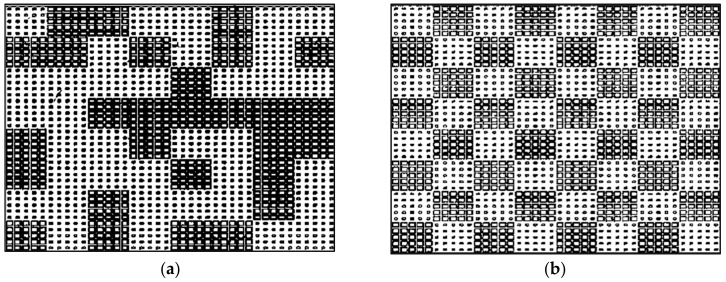
(**a**) Distribution of elements 0 and 1 in a coding diffusion metasurface; (**b**) Distribution of elements 0 and 1 in a chessboard metasurface.

**Figure 7:**
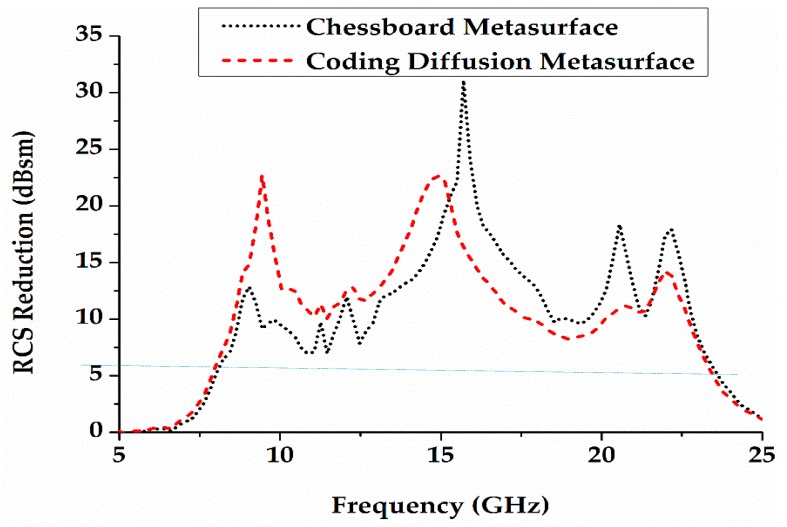
Mono-static radar cross-section (RCS) reduction of a chessboard and coding diffusion metasurface.

Figure 6 shows the schematic diagrams of the coding diffusion and chessboard metasurfaces. The total size of the metasurface is 264 mm × 264 mm × 3 mm (0.15λ × 13.4λ ×13.4λ at 15.4 GHz). The metasurface is simulated in CST microwave studio for the scattering analysis. The peak assessment of scattered intensity with a PEC plate of the same size shows the ability of RCS reduction. Good RCS reduction is achieved in the band from 8.6 GHz to 22.5 GHz for the coding diffusion metasurface, as shown in Figure 7. The simulated RCS reduction is 92% bandwidth for a coding diffusion metasurface and the maximum RCS reduction is more than 23 dB at 15.4 GHz. The traditional chessboard metasurface has high scattering field at 10.2 GHz and 11 GHz as compared to the coding diffusion metasurface. The traditional chessboard metasurface achieves a RCS reduction from 13.8 GHz to 22.5 GHz with 60% bandwidth, while the coding diffusion metasurface has the RCS reduction within a wider bandwidth, i.e., from 8.6 GHz to 22.5 GHz. It is clearly shown that the optimized coding diffusion metasurface has a wide bandwidth of 92%, as mentioned in Table 1. 

**Figure 8:**
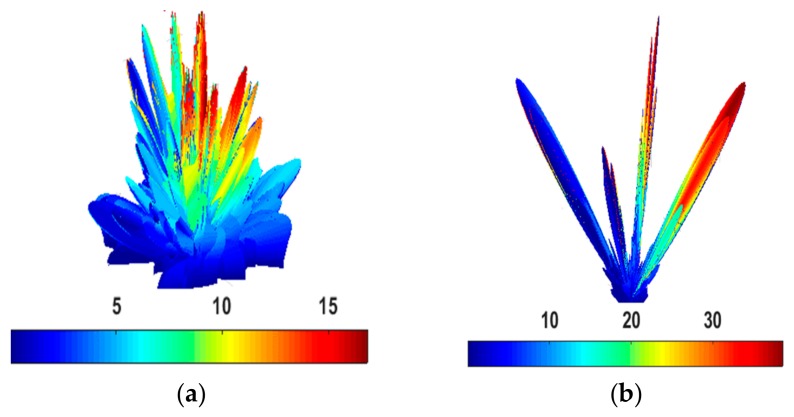
(**a**) Coding diffusion metasurface three-dimensional (3-D) plot of scattering field; (**b**) Chessboard metasurface 3-D plot of scattering field.

The chessboard and coding diffusion metasurface at 15.4 GHz are simulated, as shown in Figure 8. The backscattering is reduced significantly in the coding diffusion metasurface due to the phase cancellation of the reflected field from the unit cells 1 and 0. There are main eight scatter lobes of the coding diffusion metasurface, while the chessboard metasurface has only four main lobes as shown in Figure 8. These are summarized in Table 2. 

**Figure 9:**
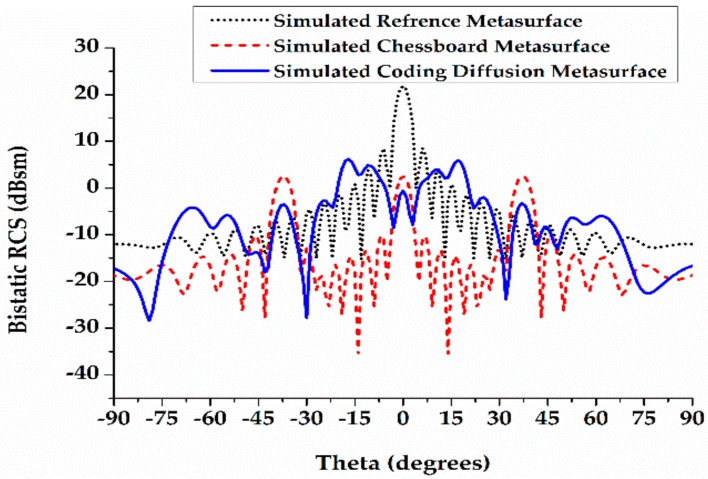
Bi-static RCS reduction of a chessboard and coding diffusion metasurface.

For comparison of the scattered field along the plane with a maximum bistatic RCS, the planes j = 0° and 40° at 15.4 GHz are shown in Figure 9. The chessboard and coding diffusion metasurface RCS are compared with a PEC surface of the same size. At the plane j = 0°, the bistatic RCS reductions of the chessboard and coding diffusion metasurfaces are 2 dB and −4 dB, respectively; the RCS reductions of the chessboard and coding diffusion metasurfaces are 19 dB and 25 dB, respectively. At the plane j = 40°, the bistatic RCSs of the chessboard and the coding diffusion metasurfaces are 3 dB and −5 dB, respectively; the RCS reductions of the chessboard and coding diffusion metasurfaces are 18 dB and 26 dB, respectively. 

The different optimization techniques have been exploit in the use of the generation of the 2D code. However, these techniques are expensive in terms of resources and speed. Employing the optimisation technique of FPGA (field programmable gate array), Coding, and Digital metamaterial, the hardware is used to generate the bits 0 and 1. It defines the array factor and after the combination of array factor with an element pattern a 2D-code is generated. Overall, the random optimisation technique that we propose in this work has a smaller number of iterations as compared to other similar works while using different techniques. The PSO (Particle swarm optimisation), Genetic algorithm, Ergodic Algorithm, and other 01/10 coding metasurface have a large number of iterations, which becomes less efficient in terms of speed from random optimisation algorithm. These algorithms normally take 300–400 iterations, while the random optimisation algorithm takes 64 iterations. The performances from Ref. [9,12,15,22,24] are verified in the dimensions, frequency band, bandwidth, and optimization technique. The proposed metasurface shows competitive potential in a wide band operation.

## 4. Fabrication and Measurement

**Figure 10:**
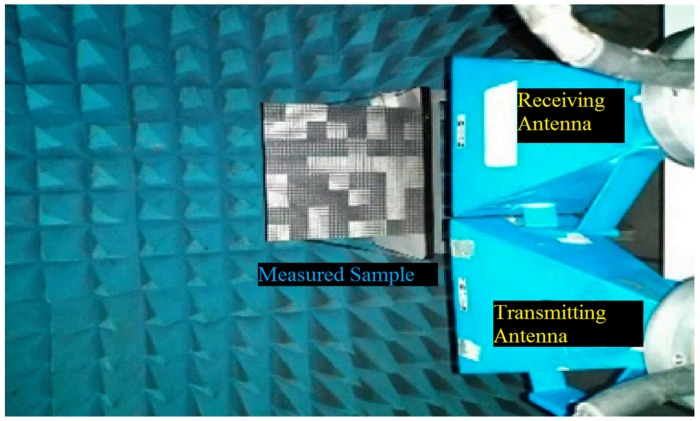
Experimental setup.

The proposed wideband metasurface with a low reflection property is experimentally demonstrated in Figure 10. The coding diffusion metasurface occupies a size of 264 mm × 264 mm with 8 × 8 tiles. It is fabricated on a substrate with a dielectric constant of 2.65 and tangent loss of 0.001. All of the measurements are performed inside an anechoic chamber. Figure 10 shows the testing environment. 

**Figure 11:**
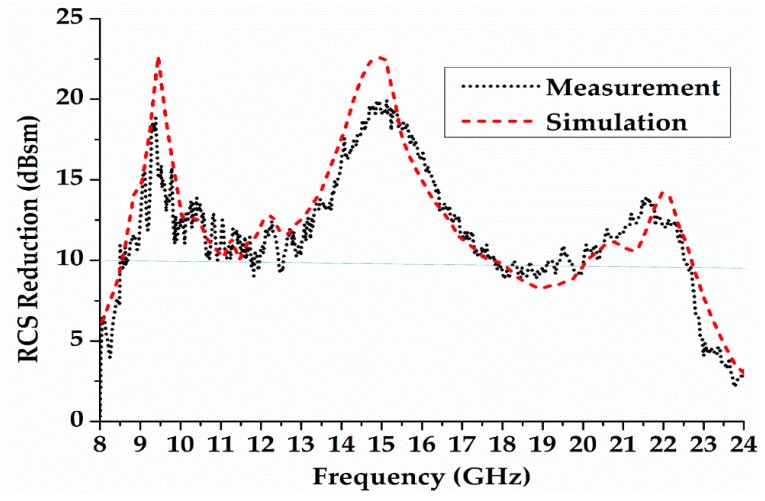
Measurement and Simulation Results of RCS Reduction.

The two horn antenna (used as transmitter and receiver) are connected with a vector network analyser (Angilent, Palo Alto, CA, USA) to measure the RCS of the designed metasurface. In this setup, the reflections from the surface of the designed metasurface are captured by the receiver. The backscattering of a metal plate (of the same dimensions as the metasurface) is measured to show the performance of RCS reduction. Figure 11 shows the simulation and measurement result of a monostatic RCS. 

The results show that the coding diffusion metasurface achieves a RCS reduction better than 9.5 dB from 8.6 GHz to 22.5 GHz (cf. Figure 11). It also shows that the measured and simulated results have good agreement. The slight discrepancies are due to manufacture tolerance and the misalignment of the horn antenna on the metasurface during the measurements. 

## 5. Conclusions

In this paper, RCS reduction has been achieved by using the coding diffusion metasurface, which is optimized through a random optimization algorithm. The unit cell is arranged according to the 2-D code that is generated from the optimisation algorithm. The difference between the reflection phases of the unit cells ranges from 143° to 217°. The simulated result demonstrates the superiority of the proposed coding diffusion metasurface to the chessboard metasurface. The proposed coding diffusion metasurface is able to achieve low RCS with a more efficient optimization algorithm over a wide bandwidth from 8.6 GHz to 22.5 GHz (92% relative bandwidth).

## Figures and Tables

**Table 1:** Comparison of the designed metasurface with previously reported works.

Similar Work	Size of Metasurface (mm^3^)	Frequency Range (<−10 dB)	Bandwidth (%)	Optimization Technique
[9]	180 × 180 × 3.5	9.4–23.28	85	Chessboard Metasurface
[12]	280 × 280 × 1.694	7–16	63	Coding and Digital Metamaterial
[15]	192 × 180 × 4	6–14	80	PSO Algorithm
[22]	328 × 328 × 3	5.4–7.4	67	Ergodic Algorithm
[24]	400 × 400 × 3.5	6–14	80	01/10 coding metasurface
This work	264 × 264 × 3	8.6–22.5	92	Random optimization Algorithm

**Table 2:** Comparison between chessboard and coding diffusion metasurface.

Technique	Size (mm^3^)	Bandwidth	Scattering Field Lobes (Dispersion of Incident Wave)	Monostatic RCS Reduction	Bistatic RCS Reduction at j = 0°/40° (15.4 GHz)	Operating Band
Designed metasurface	264 × 264 × 3	92%	Eight lobes	9	25 and 26 dB	8.6–22.5 GHz
Chessboard metasurface	264 × 264 × 3	60%	Four Lobes	7.5	19 and 18 dB	13.8–22.5 GHz
